# A New Strategy for Identification of Highly Conserved microRNAs in Non-Model Insect, *Spodoptera litura*

**DOI:** 10.3390/ijms13010612

**Published:** 2012-01-09

**Authors:** Lu Gao, Hongliang Zuo, Keling Liu, Haiyi Li, Guohua Zhong

**Affiliations:** Laboratory of Insect Toxicology, South China Agricultural University, Guangzhou 510642, China; E-Mails: gaolu0424@163.com (L.G.); zhlzuo@163.com (H.Z.); liuwkwl@126.com (K.L.); oceanyili@163.com (H.L.)

**Keywords:** microRNA, precursor, identification, stem-loop RT-PCR, non-model insect, sequence conservation, *Spodoptera litura*

## Abstract

The indigenous small non-coding RNAs, known as microRNAs (miRNAs), are important regulators of gene expression and many of them are evolutionarily conserved. Whether stem-loop RT-PCR, as a sensitive method, could be utilized to clone conserved miRNAs from non-model insects lacks information. Here, three miRNAs, *sli-miR-14*, *sli-miR-2a* and *sli-bantam*, were cloned from *Spodoptera litura* by stem-loop RT-PCR. Two groups of primers were designed, and one of them performed especially well and proved stable. The sequences of two highly conserved miRNAs, *sli-miR-14* and *sli-miR-2a* were identical to those in *Drosophila melanogaster.* To validate the reliability of this strategy, *pre-miR-14* and *pre-miR-2a* in *S. litura* as representatives were given as well; this shared high homology with those in *D. melanogaster* and *Bombyx mori*, and both mature sequences of *sli-miR-14* and *sli-mi*R-2a in their precursors shared 100% identity to the results shown by stem-loop RT-PCR. Moreover, expression patterns of these miRNAs were investigated by real-time quantitative PCR. *Sli-miR-14* and *sli-miR-2a* could be detected successfully and their expression patterns showed similar characteristics with those in model insects, further suggesting stem-loop RT-PCR technology can be used for identification of highly conserved miRNAs in non-model insects. These results provide a simplified and efficient strategy for studying the structure and function of highly conserved miRNAs, especially some critical miRNAs in non-model insects.

## 1. Introduction

MicroRNAs (miRNAs) are small (~22 nucleotides) non-coding RNAs molecules bound to 3′-untranslated regions (3′-UTR) of messenger RNA (mRNA) of the target genes by incomplete base-pairing and regulate gene expression at post-transcriptional level [1–3]. The genes encoding miRNAs are usually located in intergenic regions, some in the introns of known genes, and even within expressed sequence tags (ESTs). In the nucleus, a stem-loop (hairpin) primary miRNA (pri-miRNA) as a newly-transcribed RNA is processed into a 70~90 nt hairpin long precursor miRNA (pre-miRNA) by Drosha nuclease. Then, pre-miRNA is exported to the cytosol and cut into a short double-stranded RNA by the Dicer nuclease, which is loaded onto a protein complex called the RNA-induced silencing complex (RISC), where a protein in the Argonaute family binds to one mature strand to form an active miRNA-RISC complex, then the mature short miRNAs act on their targets and lead to the inhibition of translation [4,5].

MiRNAs have been shown to be ubiquitous in multicellular organisms and many of them are evolutionarily conserved [6–8]. It has been verified that miRNAs play pivotal roles in a wide variety of biological processes including developmental timing, cell proliferation, apoptosis and neural development [2,9,10]. MiRNAs were originally isolated by a direct cloning procedure in *Drosophila melanogaster* and many of them are sequence conserved with those of *Caenorhabditis elegans* [11–13]. In recent years, research on insects’ miRNAs has extended gradually from *D. melanogaster* to other model insects, such as *Apis mellifera* [14], *Bombyx mori* [15,16] and *Tribolium castaneum* [17]. Although rapid progresses have been achieved in discovering new miRNAs and exploring their biological roles in model insects, studies on miRNAs in non-model insects, especially in agricultural insects make slow progress.

The identification and quantification of miRNAs has been limited by their short length and low abundance. Currently, there are three main strategies for the amplification of miRNAs. Firstly, 5′ and 3′ adaptors are ligated to small RNAs, cDNA are generated by reverse transcription (RT) with primers derived from the two adaptors [18]. It can be used for massive separation and identification of miRNAs, as well as the discovery of novel miRNAs. However, complicated procedures, rigorous operation and massive work have hampered its availability and development. Secondly, only 3′ adaptor is ligated to small RNAs, miRNA-specific primer and 3′ adaptor primer are in conjunction with cDNA for miRNA amplification [19,20]. Although the phosphorylation and ligation have been omitted in this method, the whole procedure is complicated. The application of commercial kit increases the success rate, but increases the cost. Thirdly, a miRNA-specific stem-loop primer is used for RT procedure. PCR reverse primer is based on the stem-loop RT primer, while the forward primer is designed by the 5′ end of mature miRNA [21,22]. Only total RNA should be extracted from organisms, tissues or cells by this method, which provides a simple operation and costs less. Compared with the first way, designs of PCR forward primer are limited by the sequence of known miRNA in the second and third ways. Therefore the latter two are always used for quantitative determination of known miRNAs in model insects; however, until now there has been no report on the application of them for identifying miRNAs in non-model insects.

Three known miRNAs, *miR-14*, *miR-2a* and *bantam*, play important roles in the developmental stages in *D. melanogaster* [23,24], such as regulating steroid hormone signaling [25] and apoptosis [12,26,27]. Here, these three miRNAs, which exist in model insects, were presented as a model to confirm the adaptability to identify the homologues of known miRNAs in non-model insects by stem-loop RT-PCR technology. The homologues of *miR-14*, *miR-2a* and *bantam* were cloned from *Spodoptera litura*, a prevalent agriculture pest in China, by stem-loop RT-PCR, and their expression patterns in different developmental stages were also investigated to confirm the results of sequence analysis. These observations provide a new strategy for the identification of miRNAs in various organisms.

## 2. Results and Discussion

### 2.1. Amplification and Identification of *sli-miR-14, sli-miR-2a* and *sli-bantam* from *S. litura*

To identify the availability of stem-loop RT-PCR technology for cloning conserved miRNAs from non-model insects, the sequences of *miR-14*, *miR-2a* and *bantam* in model insects were searched from miRBase (Table S1). Two groups of primers for amplification were designed based on the sequences of mature miRNAs in *D. melanogaster* (shown in Experimental Section).

**Figure 1:**
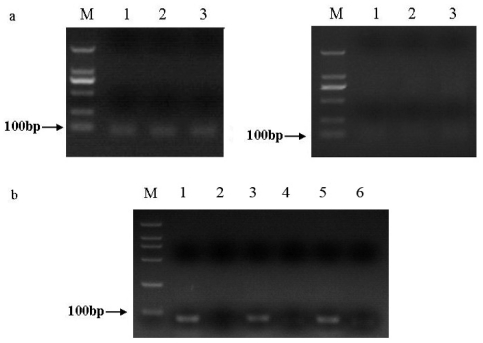
(**a**) Performance of Groups 1 primers by stem-loop RT-PCR. The bands size is amplified as shown by the arrow along with a 100 bp DNA marker (**M**). Left are PCR products of putative *miR-2a* (1), *bantam* (2), and *miR-14* (3) of *S. litura* which were 89 bp, 89 bp, 86 bp, respectively, and their corresponding minus-RT control are shown on the right; (**b**) Performance of Groups 2 primers by stem-loop RT-PCR. The bands size is amplified as shown by the arrow along with a 100 bp DNA marker (**M**). PCR products of putative *miR-2a* (1), *bantam* (3), and *miR-14* (5) of *S. litura* were 79 bp, 79 bp, 76 bp, respectively, and minus-RT control by using primers of *miR-2a* (2), *bantam* (4), and *miR-14* (6) were shown besides the putative miRNAs.

The putative homologue of the three miRNAs in *S. litura*, namely *sli-miR-14*, *sli-miR-2a* and *sli-bantam* were cloned by a stem-loop RT-PCR technique. Ten positive clones of each sample were sequenced. When Group 1 was used for amplification of *miR-14*, *miR-2a* and *bantam*, PCR products were 86 bp, 89 bp, 89 bp, respectively (Figure 1a); when Group 2 was used, PCR products were 76 bp, 79 bp and 79 bp in length, respectively (Figure 1b). Sequence analysis of the single bands showed that specific amplifications consisted of all bases of putative miRNA, PCR forward primer and reverse primer. Sequences of these cloned miRNAs in *S. litura* showed 100% match to those in *D. melanogaster*.

To verify the present results, fragments appeared in blank control group were also purified from 2.5% agarose gels, ligated into the T-vector and then sequenced. These non-specific fragments amplified with Group 1 primers were diverse according to agarose gel electrophoresis analysis, sequence analysis showed that all fragments just consisted of PCR forward primer and reverse primer, rather than the target miRNAs (Figure 1a). By using primers of Group 2, the fragments in blank control group of each miRNA displayed a single band by agarose gel electrophoresis (Figure 1b). Similarly, sequence analysis showed these fragments in each experimental group just included the forward primer, reverse primer, and/or other non-specific bases.

Performances of the two groups of primers were also different in the experimental group. Some non-specific amplifications appeared in experimental group with primers of Group 1, but the majority of sequenced colonies contained all bases of putative miRNA. By using primers of Group 2, agarose gel electrophoresis showed amplified fragment was a single band when cDNA was added into the reaction volume, sequence analysis also confirmed it. All indicated that primers bound to cDNA much more tightly than themselves in experimental group. Although two groups of primers could be used for cloning conserved miRNAs from *S. litura*, Group 2 performed better and was more stable; same results were shown in electrophoretogram (Figure 1). Based on the electrophoresis and sequence analysis of RT-PCR, Group 2 primers were more suitable for a quantitative PCR (qPCR) assay.

### 2.2. Cloning and Analysis of *pre-miR-14* and *pre-miR-2a* from *S. litura*

**Figure 2:**
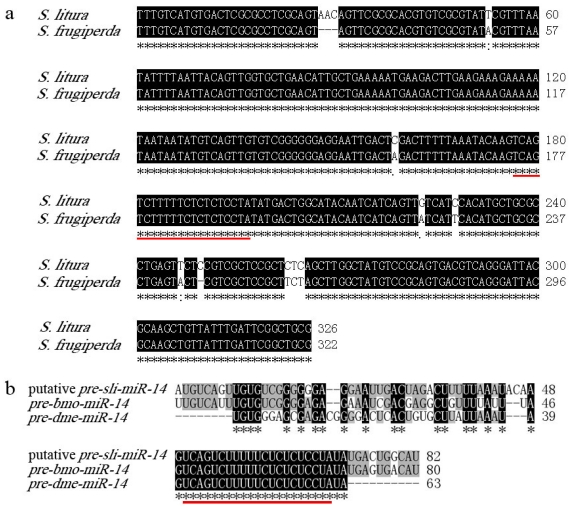
(**a**) The sequence alignment of partial sequences of *pri-miR-14* between *S. litura* and *S. frugiperda*; (**b**) The sequence alignment of entire sequences of *pre-miR-14* among *S. litura*, *B. mori* and *D. melanogaster*. Black shades represent completely conserved bases. Mature sequence of *sli-miR-14* was underlined by red line.

To verify the present results, homology of *pre-miR-14 and pre-miR-2a* were cloned from *S. litura.* According to the two ESTs of *Spodoptera frugiperda* which are highly homologous to putative *sli-miR-14* and four ESTs of *Heliothis virescens*, a 322 bp fragment was amplified using degenerate primers Pre-14F and Pre-14R from genomic DNA of adult of *S.litura*. This fragment showed 97.5% identity to the ESTs of *S. frugiperda* and 82 bp putative *pre-sli-miR-14* was contained in it (Figure 2a). Blast procedure had been performed to analyze the homology among the putative *pre-miR-14* in *S.litura* and that in *B. mori* and *D. melanogaster.* Results showed the putative *pre-sli-miR-14* shared 83.8% and 73% homology to *pre-bmo-miR-14* and *pre-dme-miR-14* respectively (Figure 2b).

**Figure 3:**
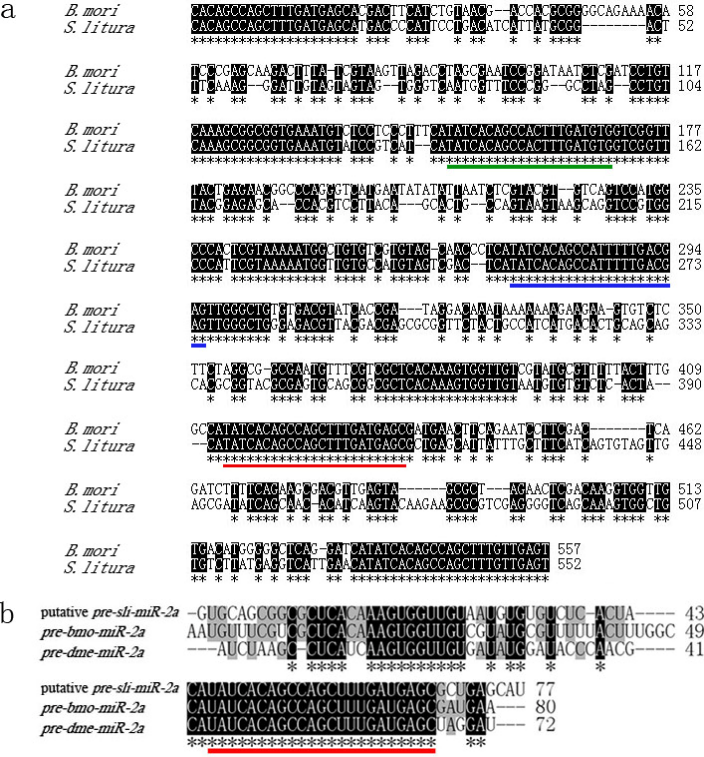
(**a**) The sequence alignment of partial sequences of *pri-miR-2a* between *S. litura* and *B. mori*; (**b**) The sequence alignment of entire sequences of *pre-miR-2a* among *S. litura*, *B. mori* and *D. melanogaster*. Black shades represent completely conserved bases. Mature sequence of *sli-miR-2a* was underlined by red line, putative *sli-miR-13a* and *sli-miR-13b* were also underlined by green and blue line, respectively.

A cluster of *miR-2a-1*, *miR-2a-2*, *miR-2b*, *miR-13a* and *miR-13b* is localized on chromosome 1 of *B. mori*, indicating there are two precursors of *miR-2a*, and *miR-2a-2* is in the interior of this cluster. Therefore, Primers Pre-2aF and Pre-2aR were designed according to the conserved miRNAs for amplifying *pre-miR-2a-2*, and rising nealing temperature to 65 °C to inhibit non-specific amplification. A 552bp fragment containing putative *pre-sli-miR-2a-2* was amplified from genomic DNA of adult of *S. litura*. Sequence analysis showed that this fragment was 68.9% identity to the homologue in *B. mori*, there are three highly conserved regions besides two primers’ sites, where highly conserved miRNAs including *miR-2a-2*, *miR-13a* and *miR-13b* exist (Figure 3a). The putative *pre-sli-miR-2a* showed 81.1% and 70.4% similarities with *pre-bmo-miR-2a* and *pre-dme-miR-2a* respectively (Figure 3b).

**Figure 4:**
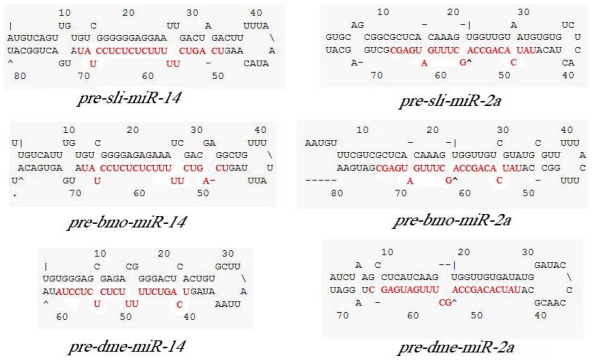
The stem-loop structures of precursors and mature forms (highlighted in red) of *miR-14* (**a**) and *miR-2a* (**b**) in *S. litura*, *B. mori* and *D. melanogaster*.

Moreover, both mature sequences of *miR-14* and *miR-2a* in their precursors shared 100% identity to the results shown by stem-loop RT-PCR. In order to verify the result, secondary structure of two putative *pre-miRNAs* were predicted by mfold program. Results showed that putative *pre-miR-14* and *pre-miR-2a* of *S. litura* can form stable stem-loop structures (initial Δ*G* = −41.80 and Δ*G* = −41.20 respectively) and are highly homologous to those of *B. mori* and *D. melanogaster*, indicating they are the precursors of *sli-miR-14* and *sli-miR-*2a (Figure 4). Above all suggested stem-loop RT-PCR could be used in identification of highly conserved miRNAs in non-model insects.

### 2.3. Expression Patterns of *sli-miR-14, sli-miR-2a* and *sli-bantam* from *S. litura*

The expression patterns of the putative miRNAs, *sli-miR-14*, *sli-miR-2a* and *sli-bantam* were also studied to make a further validation of this method. Group 2 primers were used for a qPCR assay. Although putative *sli-bantam* could be cloned by stem-loop RT-PCR, no stable results of qPCR could be obtained here (data not shown). However, the expression patterns of *sli-miR-14* and *sli-miR-2a*, two highly conserved miRNAs in insect, could be obtained in this assay. PCR efficiency was determined on the miRNAs as well as 5S rRNA. The efficiency of *miR-14*, *miR-2a* and 5S rRNA was close to the ideal value of 2 (Table 1), therefore relative quantification (RQ) of miRNA expression was calculated with 2^−Δ ΔCt^ method [28,29].

**Figure 5:**
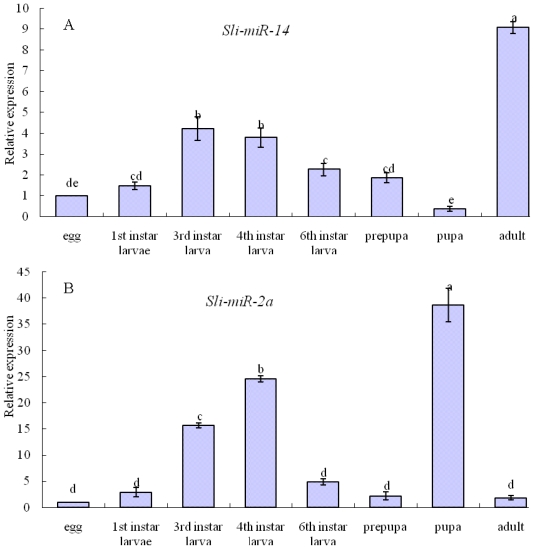
The relative expression levels of *sli-miR-14* (**a**) and *sli-miR-2a* (**b**) in different developmental stages. Data are presented as means ± SE for three experimental replicates. Letters above each bar indicate expression significance in the different stages by Duncan’s Multiple Ranges Test.

*Sli-miR-14* was expressed strongly in all developmental stages of *S. litura*, but there were some differences among developmental stages. The relative expression levels of *sli-miR-14* were 1.47-, 4.22-, 3.78-, 2.26-, 1.87-, 0.37- and 9.07-fold higher in the first instar, third instar, fourth instar, six instar, pre-pupa, pupa and adult than in eggs, respectively (Figure 5a), which indicated that *sli-miR-14* expressed in pupal stage lower than in other stages, and especially strongly in adult stage.

Development-specific expression patterns for *sli-miR-2a* were also detected by qPCR. There were significant differences among various developmental stages, the relative expression levels of *sli-miR-2a* were 2.89-, 15.65-, 24.55-, 4.88-, 2.23-, 38.68- and 1.81-fold higher in the first instar, third instar, fourth instar, six instar, prepupa, pupa and adult than in egg, respectively (Figure 5b). *Sli-miR-2a* expressed at low level in egg, prepupa and adult stages, instead expressed most strongly in pupa stage, which accord with that in model insects, *D. melanogaster* [30] and *B. mori* [24].

### 2.4. Discussion

**Figure 6:**
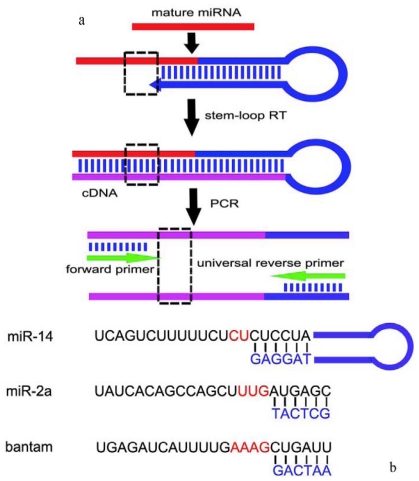
The principle of stem-loop RT-PCR in present paper: (**a**) Stem-loop primer was designed to increase the length of mature miRNAs. PCR forward primer was designed by putative miRNAs, the universal reverse primer was designed by stem-loop primer. The box formed by dotted line represents the bases not included in primers; (**b**) Six nucleotides of 3′ end of stem-loop RT primer are complementary with 3′ end of mature miRNAs of *D. melanogaster*. Two bases, three bases and four bases were not included in RT-PCR primers of *miR-14*, *miR-2a* and *bantam*, respectively (colored red), thus miRNAs or non-specific amplification could be distinguished by sequence analysis.

As we know, stem-loop RT-PCR is a very sensitive method for quantitative determination of known miRNAs, even one base difference between two miRNAs in the same family could be detected and distinguished by this method [31]. Whether stem-loop RT-PCR could be used for identifying conserved miRNAs in non-model insects was studied in this paper. Two groups of primers were designed to access the availability of this method. Six nucleotides of 3′ end of stem-loop RT primer were complementary with 3′ end of mature miRNAs, other nucleotides of RT primer were partially complementary with themselves to form a stem, and the remaining nucleotides form a loop. The PCR reverse primer was universal, while the forward primer was designed by mature miRNAs (Figure 6a). In order to make a distinction between specific and non-specific amplifications, only partial bases of miRNA were contained in forward primer. Two bases, three bases and four bases were not included in primers of *miR-14*, *miR-2a* and *bantam*, respectively (Figure 6b). The designing of stem-loop RT primers often presents learners with difficulties in some way; the primers designed in this assay also provide references for later research on other miRNAs.

*MiR-14* and *miR-2a* are more conserved members of miRNAs family than *bantam* in model insects including *D. melanogaster* and *B. mori* (Table S1). Sequence analysis indicated that homologue of the three miRNAs could be cloned from *S. litura* by stem-loop RT-PCR. In order to confirm whether the PCR products are endogenous miRNAs, putative precursors of *miR-14* and *miR-2a* as representatives were also cloned from *S. litura*, results showed that both their sequences and secondary structures were highly conserved with model insects. The mature *sli-miR-14* and *sli-miR-2a* in their precursors shared 100% identity to the results shown by stem-loop RT-PCR. Then, *sli*-m*iR-14* and *sli*-*miR-2a* were successfully detected by a qPCR assay and their expression patterns showed similar characteristics with those in model insects. Instead, abnormal amplification appeared in qPCR of *sli*-*bantam*, there is no significant difference in Ct values in the presence or absence of cDNA added to the RT reactions (data not shown). As mentioned above, only six bases of 3′ end of putative miRNA were complementary with stem-loop RT primer, but PCR forward primer was restricted by the 5′ end of miRNAs, suggesting this strategy can be used for identification of highly conserved miRNAs and distinguished variation appeared in 3′ region of miRNAs. However, this method is difficult to differentiate variation appeared in 5′ region of miRNAs. Some miRNAs with evolutional variation in their 5′ region, such as *bantam*, cannot show accurate sequences in non-model insects and toned to be identified together by other methods.

Although the identification of miRNAs in non-model insects with stem-loop RT-PCR is limited by 5′ region of putative miRNA, large amounts of miRNAs that are highly conserved, such as *miR-14* and *miR-2a*, can be simply cloned by this method. It has been clearly stated that cell autonomous anti-apoptotic activity mediated by *miR-14* and *miR-2a* [12,27], *miR-14* also plays critical role in molting process [25], indicating miRNAs are involved in strict developmental regulation in insects. Studies on miRNAs in various insects, especially agricultural pests, may provide new ideas for pest control. However, so many difficulties stand in our way for identifying miRNAs in non-model insects, a simple and efficient method is urgently necessary.

Applying stem-loop RT-PCR in identification of highly conserved miRNAs from *S. litura* provides a new strategy for studying miRNAs in a more extensive field. As mentioned above, direct cloning is more appropriate for the cloning of massive miRNAs and the discovery of novel miRNAs, this powerful but blind task produces a huge workload. With the help of computer programs, researchers can make an easy prediction on miRNAs in the genomic database of model insects, and then make a direct cloning according to the predicted results. Unfortunately, this has no advantages for non-model insects. Currently, most of the studies on miRNAs of non-model insects have focused on the direct cloning of miRNAs, however, the cloned miRNAs mostly are conserved; many even have identical sequences with those in model insects. Compared with direct cloning, stem-loop RT-PCR has a clearly defined aim for cloning selected conserved miRNAs, which simplifies operation and saves cost. Meanwhile, the discovery of massive miRNAs in model insects gives us lots of resources for further study in more extensive field. When one or a few important and highly conserved miRNAs were expected to research in non-model insects, this strategy greatly improves efficiency.

## 3. Experimental Section

### 3.1. Sample Preparation

A laboratory strain of *S. litura* was purchased from the Entomology Institute of SUN YAT-SEN University, Guangzhou, China. Insects were routinely reared at 25 ± 1 °C and 70 ± 10% relative humidity under a 16:8 h (light:dark).

*S. litura* ovary cell lines SL-1 were routinely maintained at 27 °C at the laboratory. SL-1 cells were cultured in insects’ Grace medium, supplemented with 7% fetal bovine serum (FBS) (Gibco, USA), 0.3% yeast extract (Oxoid, UK) and 0.3% lactalbumin hydrolysate (Oxoid, UK). Culture medium was changed every 3 days, and subcultured SL-1 cells every 6 days.

Eight samples including eggs, larvae (selected first, third, forth, sixth instar as representatives), pre-pupae, pupae and adults were collected and stored at −80 °C for total RNA extraction. The third-instar larvae and SL-1 cells were prepared for stem-loop RT-PCR, and samples of eight stages were prepared for real-time qPCR.

### 3.2. Stem-Loop RT-PCR Primers Designing

Two groups of primers including stem-loop primer and corresponding PCR primers were designed based on the mature sequences of the three miRNAs in *D. melanogaster* (Table S1). The Group 1 primers were designed by previous reports [15], Group 2 primers haven’t reported before. The partial bases of stem-loop RT primer of three miRNAs were universal, Group 1 was: 5′-GTCGTATCCAGTGCAGGGTCCGAGGTATTCGCACTGGATACGAC-3′ (Table 2), Group 2 was: 5′-GCACTTCAGTGTCGTGGTCAGTGACGGCAATTTGAAGTGC-3′ (Table 3). Six bases of 3′ end of stem-loop RT primer were complementary with 3′ end of mature miRNA, and the PCR forward primer was paired with 5′ end of mature miRNA (principle in Figure 3).

### 3.3. RNA Extraction and Reverse Transcription

The extractions of total RNA from all stages of *S. litura* and SL-1 cells were performed by using TRIzol kit (Invitrogen) according to the manufacturer’s instructions. cDNA was synthesized from total RNA with miRNA-specific stem-loop RT primer. Reverse transcriptase reactions contained 2 μg of RNA samples, 125 nM stem-loop RT primer, 1× RT buffer, 2.5 mM each of dNTPs (TaKaRa), 200 U·μL^−1^ PrimerScript Reverse Transcriptase (TaKaRa) and 40 U·μL^−1^ RNase Inhibitor (TaKaRa). The 11 μL reactions consisting of total RNA, stem-loop RT primer and ddH_2_O was first incubated for 10 min at 70 °C, 2 min for ice-bath, and other reagents were mixed together after a transient centrifugation. These 25 μL reactions were incubated in a MyCycler Thermal Cycler (Bio-Rad, Hercules, CA) for 60 min at 42 °C, 15 min at 72 °C, and then held at 4 °C.

### 3.4. PCR of *sli-miR-14, sli-miR-2a* and *sli-bantam*

PCR was performed in a 50 μL reaction volume containing 1 μL cDNA, 10 μM of each primer, 10× PCR buffer, 2.5 mM each of dNTPs (TaKaRa) and 5 U·μL^−1^ Taq DNA polymerase (TaKaRa). The condition for the PCR was 94 °C for 3 min, followed by 35 cycles of 94 °C for 30 s, 55~60 °C for 30 s, 72 °C for 30 s, and additional polymerization step at 72 °C for 10 min. Target fragments were purified from 2.5% agarose gels and ligated into the T-vector (TaKaRa) and then sequenced.

### 3.5. Pre-miRNA Primers Designing

Two gene fragments of *S. frugiperda* (FP355748.1 and FP352735.1) were found in EST Database (NCBI) by homologous searching according to the sequence of pupative *sli-miR-14*. Partial sequence of the two ESTs showed high similarities with *pre-bmo-miR-14* and *pre-dme-miR-14*. Then four gene fragments of *Heliothis virescens* (GT205268.1, GT212851.1, GT204572.1 and GT204571.1) was found according to the two ESTs of *S. frugiperda*. Based on these homologous sequences, degenerate primers Pre-14F and Pre-14R were designed for cloning *pre-miR-14* from *S. litura.* Pre-14F: TTTGTC ATGTGACTCGCGCC; Pre-14R: CGCAGCCGAATCAAATAACA.

By searching *bmo-miR-2a* in Silkworm Genome Database, it is noticed that there are two precursors of *pre-bmo-miR-2a* which localized on chromosome 1 and organized as a cluster with *pre-miR-2b*, *pre-miR-13a* and *pre-miR-13b* (Figure 5). Based on the conserved character of miRNA, Pre-2aF and Pre-2aR for cloning *pre-miR-2a-2* were designed according to the mature sequences of *miR-2b* and *miR-2a-1* which located at both ends of the cluster. Pre-2aF: ACTCAACAAAGCTGGCTGTGA; Pre-2aR: CACAGCCAGCTTTGATGAGC.

### 3.6. Genomic DNA Isolation and the Amplification of *pre-miR-14* and *pre-miR-2a* from *S. litura*

Total genomic DNA was isolated from the adult of *S. litura* according to the instruction of E.Z.N.A. Insect DNA Kit (OMEGA, USA). The PCR reaction was performed with the following protocol: 94 °C for 3 min; followed by 35 cycles of 94 °C for 30 s, 55~65 °C for 30 s, 72 °C for 45 s, and additional polymerization step at 72 °C for 10 min. Amplified DNA was purified by 2% agarose gels, and ligated into T-vector (TaKaRa), then positive clones were sequenced.

### 3.7. Real-Time Quantitative PCR

The cDNAs were diluted 10 times to perform PCR for expression confirmation or qPCR for expression patterns analysis. The 25 μL reaction volume consisting of 2 μL cDNA, 12.5 μL SYBR Green (TaKaRa), 9.5 μL ddH_2_O, 0.5 μL of forward primer (10 μM) and 0.5 μL of reverse primer (10 μM) for each miRNAs. The reactions were performed on BIO-RAD CFX96 Real-Time PCR Detection System (Bio-Rad, Hercules, CA) following the manufacturer’s recommendations. The optimized real-time PCR program was 94 °C for 20 s, followed by 40 cycles of 95 °C for 10 s, 60 °C for 20 s, 72 °C for 10 s. After the cycling protocol, melting curves were obtained by increasing the temperature from 70 °C to 95 °C (0.4 °C/s) to denature the double-stranded DNA. PCR products were detected by electrophoresis with 3% agarose gel containing ethidium and photographed under UV light. There has not been a standard control for expression normalization for miRNAs, thus 5S rRNA as an internal control was used in this assay [19]. The forward primer was: CCAACGTCCATACCAT GTTGA, the reverse primer was: GCGGTCACCCATCCAAGTA.

Relative quantification (RQ) of miRNA expression was calculated with 2^−Δ ΔCt^ method [29]. To experimentally determine PCR efficiency, diluted cDNAs (10^0^, 10^−1^, 10^−2^ and 10^−3^) were amplified by real-time PCR. Plots were made of the log of template concentration *versus* the CT and the PCR efficiency was calculated from the slope of the line using the equation: *E* = 10^−1/slope^.

### 3.8. Sequence and Data Analysis

Sequence analysis was performed by DNASTAR 7.0. Sequence similarity for conserved domains of miRNAs was analyzed by MIRBASE, Silkworm Genome Database and BLAST programs on the National Center for Biotechnology Information (NCBI) Multiple sequence alignments of partial pri-miRNA and entire pre-miRNA among related species were performed by ClustalW2. The pre-miRNA sequence was subjected to an RNA secondary structure check using mfold program.

## 4. Conclusion

Three homologues of known miRNAs, *miR-14*, *miR-2a* and *bantam* in model insects, were cloned from *S. litura* by stem-loop RT-PCR, and named *sli-miR-14*, *sli*-*miR-2a* and *sli-bantam. Pre-miR-14* and *pre-miR-2a* as representatives were also cloned from *S. litura;* both their sequences and secondary structures shared a high degree of homology with those in model insects, and the mature sequences of *miR-14* and *miR-2a* in their precursors shared 100% identity to the results shown by stem-loop RT-PCR. Moreover, expression patterns analysis indicated that the highly conserved miRNAs, both *miR-14* and *miR-2a*, could be detected successfully by real-time quantitative PCR, which confirms that stem-loop RT-PCR can be used not only for quantification of miRNAs, but also for identifying highly conserved miRNAs in non-model insects. All these results showed stem-loop RT-PCR is a simplified and efficient strategy for cloning highly conserved miRNAs in non-model insects, providing a new idea for further study on those highly conserved and critical miRNAs in various organisms, such as the interaction between miRNAs and their target mRNAs.

## Figures and Tables

**Table 1:** Efficiency of amplification for miRNAs and 5S rRNA.

RNA	linear equation	PCR efficiency	*R*^2^
*Sli-miR-14*	*y* = −3.3125*x* + 39.562	2.0039	0.9987
*Sli-miR-2a*	*y* = −3.2811*x* + 36.678	2.0173	0.9995
5S	*y* = −3.3915*x* + 33.219	1.9718	0.9945

**Table 2:** Sequences of stem-loop RT primers, forward primers and reverse primers of Group One.

miRNAs	Primer	Sequence (5′-3′)
*Sli-miR-14*	RT	GTCGTATCCAGTGCAGGGTCCGAGGTATTCGCACTGGATACGACTAGGAG
	Forward	CGGGGCTCAGTCTTTTTCT
	Reverse	GTGCAGGGTCCGAGGT
*Sli-miR-2a*	RT	GTCGTATCCAGTGCAGGGTCCGAGGTATTCGCACTGGATACGACGCTCAT
	Forward	GCCAGTATCACAGCCAGCT
	Reverse	GTGCAGGGTCCGAGGT
*Sli-bantam*	RT	GTCGTATCCAGTGCAGGGTCCGAGGTATTCGCACTGGATACGACAATCAG
	Forward	GCCCCGTGAGATCATTTTG
	Reverse	GTGCAGGGTCCGAGGT

**Table 3:** Sequences of stem-loop RT primers, forward primers and reverse primers of Group Two.

miRNAs	Primer	Sequence (5′-3′)
*Sli-miR-14*	RT	GCACTTCAGTGTCGTGGTCAGTGACGGCAATTTGAAGTGCTAGGAG
	Forward	CGCACGACGCATCAGTCAGTCTTTTTCT
	Reverse	GCACTTCAGTGTCGTGGTCAGTGACGGCAATT
*Sli-miR-2a*	RT	GCACTTCAGTGTCGTGGTCAGTGACGGCAATTTGAAGTGCGCTCAT
	Forward	CGACACACACCATCAGTATCACAGCCAGCT
	Reverse	GCACTTCAGTGTCGTGGTCAGTGACGGCAATT
*Sli-bantam*	RT	GCACTTCAGTGTCGTGGTCAGTGACGGCAATTTGAAGTGCAATCAG
	Forward	CGCATCGTAGCATCGCTGAGATCATTTTG
	Reverse	GCACTTCAGTGTCGTGGTCAGTGACGGCAATT
